# C5-Azobenzene-substituted 2'-Deoxyuridine-containing Oligodeoxynucleotides for Photo-Switching Hybridization

**DOI:** 10.3390/molecules19045109

**Published:** 2014-04-22

**Authors:** Shohei Mori, Kunihiko Morihiro, Satoshi Obika

**Affiliations:** 1 Graduate School of Pharmaceutical Sciences, Osaka University, 1-6 Yamadaoka, Suita, Osaka 565-0871, Japan; E-Mail: mori-s@phs.osaka-u.ac.jp; 2 National Institute of Biomedical Innovation (NIBIO), 7-6-8 Saito-Asagi, Osaka 567-0085, Japan

**Keywords:** azobenzene, molecular switch, nucleoside, oligonucleotide, photochromism

## Abstract

A new photoisomeric nucleoside **dU^Az^** bearing an azobenzene group at the C5-position of 2'-deoxyuridine was designed and synthesized. Photoisomerization of **dU^Az^** in oligodeoxynucleotides can be achieved rapidly and selectively with 365 nm (forward) and 450 nm (backward) irradiation. Thermal denaturation experiments revealed that **dU^Az^** stabilized the duplex in the *cis*-form and destabilized it in the *trans*-form with mismatch discrimination ability comparable to thymidine. These results indicate that **dU^Az^** could be a powerful material for reversibly manipulating nucleic acid hybridization with spatiotemporal control.

## 1. Introduction

Regulation of nucleic acid hybridization by some external stimuli is a rewarding challenge due to its potential to control gene expression flow from DNA to protein at a predetermined place and time. This technique could allow for spatiotemporal controllable pharmacotherapy based on nucleic acid agents. The regulation of nucleic acid hybridization is also important in the field of nanotechnology, such as in the construction of DNA-origami [1,2,3]. Modified oligonucleotides (ONs) that can reversibly alter the hybridization ability by noninvasive external stimuli are therefore necessary. The most promising external stimulus is light, due to the possibility of accurately controlling the location, dosage and time of the irradiation. For example, Asanuma *et al.* have reported reversible photoregulation of DNA duplex formation via installation of azobenzene moieties on ONs [4,5]. Azobenzene and its derivatives are commonly adopted due to their rapid photoisomerization and drastic changes in geometry and dipole moment [6,7].

**Figure 1:**
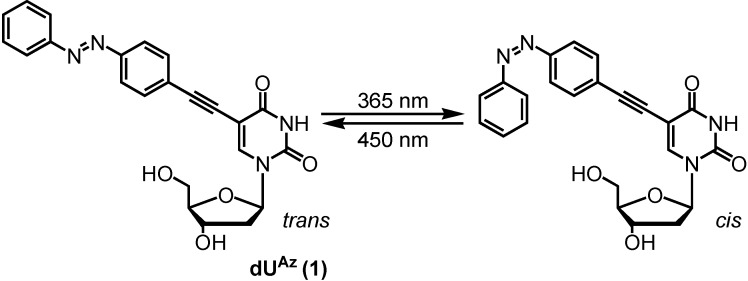
Photoisomeric nucleoside used in this study.

In this study, we describe a new type of azobenzene-modified nucleoside that reversibly changes its properties upon photoisomerization by ultraviolet (365 nm) or visible light (450 nm). There are several positions to attach a photochromic moiety to a nucleoside, and we have selected the C5 position of 2'-deoxyuridine (**dU^Az^**, Figure 1) [8]. It is predicted that the azobenzene moiety of **dU^Az^** is projected into the major groove of the double helix via a rigid ethynyl linker. We assumed that the duplexes containing *trans-***dU^Az^** would be destabilized because the hydrophobic azobenzene moiety extends to the outside of the groove [9] which surrounded by a highly polar aqueous phase, and interferes with hydration and the formation of interstrand cation bridges to stabilize the duplexes [10,11]. Meanwhile, *cis*-**dU^Az^**-modification would not affect the duplex stability due to compact conformation of the azobenzene moiety. In other words, the affinity of ONs containing **dU^Az^** for complementary single-stranded DNA or RNA may be reversibly changed, triggered by light.

## 2. Results and Discussion

### 2.1. Synthesis of **dU^Az^** Phosphoramidite and **dU^Az^**-Modified Oligodeoxynucleotides

**Scheme 1:**
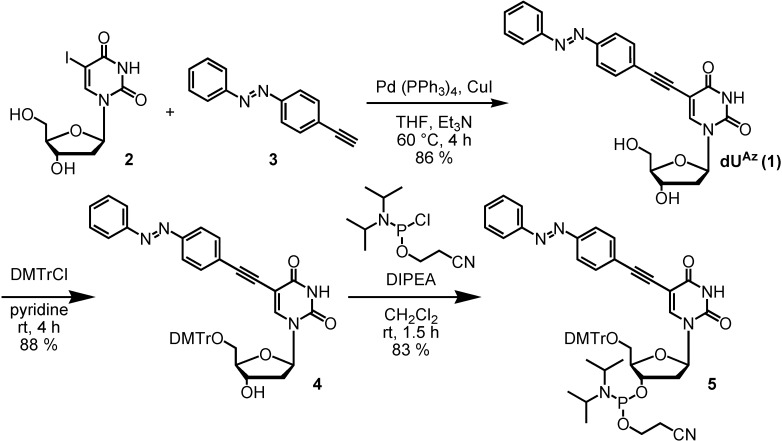
Route for the synthesis of **dU^Az^** phosphoramidite.

The synthetic route of **dU^Az^** phosphoramidite is outlined in Scheme 1. **dU^Az^** nucleoside **1** was synthesized from the corresponding 2'-deoxy-5-iodouridine (**2**) through a palladium-catalyzed cross-coupling reaction [12] with 4-ethynylazobenzene **3**[13]. Tritylation at the primary hydroxyl group of **1** with DMTrCl and phosphitylation at the secondary hydroxyl group yielded phosphoramidite **5**. The amidite **5** was incorporated into the oligodeoxynucleotide using conventional solid-phase phosphoramidite synthesis and purified by reverse-phase HPLC (29% yield). The ON sequences used in this study are shown in Table 1.

**Table 1:** The oligonucleotides used in this study.

ON	Sequence	
**6**	5'-d(GCGTTTTTTGCT)-3'	control DNA
**7**	5'-d(GCGTT**U^Az^**TTTGCT)-3'	**dU^Az^**-modified DNA
**8**	5'-d(AGCAAAAAACGC)-3'	full match DNA
**9**	5'-d(AGCAAATAACGC)-3'	mismatch DNA (T)
**10**	5'-d(AGCAAACAACGC)-3'	mismatch DNA (C)
**11**	5'-d(AGCAAAGAACGC)-3'	mismatch DNA (G)
**12**	5'-r(AGCAAAAAACGC)-3'	full match RNA
**13**	5'-r(AGCAAAUAACGC)-3'	mismatch RNA (U)
**14**	5'-r(AGCAAACAACGC)-3'	mismatch RNA (C)
**15**	5'-r(AGCAAAGAACGC)-3'	mismatch RNA (G)

### 2.2. Photoisomerization Property of **dU^Az^**

**Figure 2:**
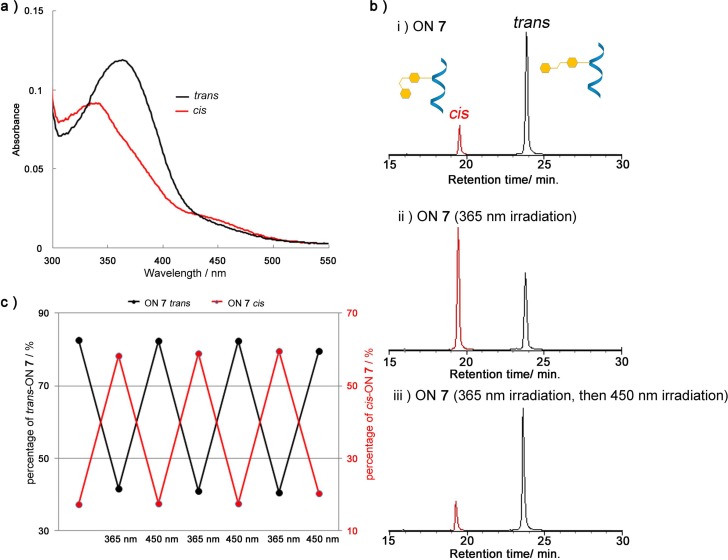
Photoisomerization properties of **dU^Az^** in oligodeoxynucleotide. (**a**) Absorbance spectra of *trans-* (black line) and *cis-* (red line) ON **7**. (**b**) HPLC analysis of the photoisomerization of ON **7**; (i) Before irradiation; (ii) after 365 nm irradiation for 10 s; (iii) subsequent irradiation at 450 nm, 10 s. (**c**) Repetitive photoisomerization of ON **7** induced by alternative light irradiation at 365 nm and 450 nm. The percentages of *trans-* (black line) and *cis-* (red line) ON **7** obtained from the HPLC peak areas are shown. Conditions: ON **7** (4.0 µM), NaCl (100mM) in sodium phosphate buffer (10 mM, pH 7.0) was irradiated at room temperature.

We initially investigated the efficiency of the **dU^Az^** *cis-trans* photoisomerization property in ON by UV spectra and HPLC analysis. UV spectra of *trans/cis* ON **7**, showed that photoisomerization of *trans*-**dU^Az^** to *cis*-**dU^Az^** decreased absorbance at 365 nm and increased absorbance at 310 nm and 450 nm (Figure 2a). The λ_max_ of *cis*-form (340 nm) was blue-shifted compared to that of the *trans*-form (365 nm), as was the case with previous reports [6,7,14]. The *trans*-form **dU^Az^** was photoisomerized to the *cis*-form by a 10-second irradiation of 365 nm monochromic light with 60% conversion, as determined by the HPLC peak areas (Figure 2b). In addition, subsequent 10-second irradiation of 450 nm yielded the *trans* form isomer with 80%. The HPLC analysis showed no side products from the reactions.

Even when the photoirradiation was repeated three times, the efficiency of the **dU^Az^** *cis-trans* photoisomerization was not attenuated (Figure 2c). It can therefore be concluded that **dU^Az^** has a rapid and highly efficient *cis-trans* photoisomerization property and the potential to work as a photo-switch for various biomolecules.

We investigated the differences in the thermal stability of 12-bp duplexes containing **dU^Az^** in the *trans-* and *cis-*forms by monitoring the melting temperature (*T*_m_) following the way of azobenzene- modified nucleoside containing ONs (Table 2) [15,16]. DNA duplex **7/8** showed a modest *T*_m _difference (Δ*T*_m_) between the *trans-* and *cis-*forms, namely, the *T*_m_ value of the *cis-*form was 2 °C higher than that of the *trans-*form. On the other hand, the ON **7**/RNA **12** duplex showed a larger *T*_m_ difference. The *T*_m_ value of the *cis*-form was 5 °C higher than that of the *trans*-form. It is noteworthy that the *cis*-ON **7**/RNA **12** duplex showed a *T*_m_ value comparable to that of natural DNA **6**/RNA **12** duplex. According to past studies, the *cis*-form photochromic moieties generically destabilize the duplex because of its interference with the vicinity bases stacking interaction [4,5,17,18,19].In this study, ON containing **dU^Az^** showed a higher hybridization ability when **dU^Az^** is *cis*-form rather than *trans*-form, unlike ONs containing the exiting photochromic nucleoside. Brown *et al.* have reported that hydrophobic buta-1,3-diynyl anthracene in ON leads to significant destabilization of the duplex, probably because the aromatic moiety is exposed to the aqueous environment [9]. The azobenzene moiety of *trans*-**dU^Az^** also would extend to the outside of the major groove, a highly polar aqueous phase. This may have an impact on the groove hydration and the formation of interstrand cation bridges, and lead to destabilization of the duplex containing *trans*-**dU^Az^**.

**Table 2:** UV-melting points of 12-bp duplexes. *^a^*

Duplex	*T*_m_ [°C]			Δ*T*_m_ [°C] *^b^* (*T*_m_ *_cis_* - *T*_m_ *_trans_*)
	*trans ^c^*		*cis ^d^*	
**6/8**		52		-
**7/8**	47		49	2
**6/12**		47		
**7/12**	42		47	5

*^a^* All *T*_m_ values for the duplexes (4.0 µM) were determined in 10 mM sodium phosphate buffer (pH 7.0) containing 100 mM NaCl. The *T*_m_ values given are the average of at least three data points; *^b^* The change in the *T*_m_ value induced by the *cis-trans* photoisomerization; *^c^* The percentage of *trans* isomer was *ca.* 80%; *^d^* The percentage of *cis* isomer was *ca.* 60%.

Finally, we investigated the mismatch discrimination ability of ON containing **dU^Az^**. The *T*_m_ values of mismatched DNA duplexes containing **dU^Az^** were found to be 14 or 15 °C lower than that of ON**7**/DNA**8** in both *trans-* and *cis*-form (Table 3). Toward complementary ssRNA, ON containing **dU^Az^** could also discriminate mismatched bases comparable to ON**7** (Table S1 in Supplementary Material). These results indicate that the mismatch discrimination ability of ON containing *trans*-/*cis*-**dU^Az^** is not spoiled by the C5-substituted-azobenzene moiety of **dU^Az^**.

**Table 3:** UV-melting points of DNA duplexes with a mismatched base pair. *^a^*

Duplex	Base pair	*T*_m_ [°C]		Δ*T*_m_ [°C] *^b^*	
		*trans ^c^*	*cis ^d^*	*trans ^c^*	*cis ^d^*
**6/9**	T:T	40		−12	
**6/10**	T:C	37		−15	
**6/11**	T:G	41		−11	
**7/9**	**U^Az^**:T	33	35	−14	−14
**7/10**	**U^Az^**:C	33	34	−14	−15
**7/11**	**U^Az^**:G	33	35	−14	−14

*^a^* All *T*_m_ values for the duplexes (4.0 µM) were determined in 10 mM sodium phosphate buffer (pH 7.0) containing 100 mM NaCl. The *T*_m_ values given are the average of at least three data points; *^b^* Δ*T*_m_ values are calculated relative to the *T*_m_ values of matched DNA **6**/DNA **8** (52 °C) or ON **7**/DNA **8** (47 °C for *trans* and 49 °C for *cis*) duplexes.; *^c^* The percentage of *trans* isomer was *ca.* 80%; *^d^* The percentage of *cis* isomer was *ca.* 60%.

We achieved synthesis of the photoisomeric nucleoside, **dU^Az^**, for which the hybridization can be controlled by using different wavelengths of light. The Δ*T*_m_ value between the *trans*- and *cis*-form is more remarkable in the DNA/RNA duplex than the DNA duplex. Although **dU^Az^** photoisomerization induced modest *T*_m_ differences, the modification of ONs with multiple **dU^Az^** units or the introduction of substituents to the azobenzene moiety [20] could enhance the Δ*T*_m_ value between the *trans*- and *cis*-forms. Our strategy indicated the possibility of photo-switches based on **dU^Az^**-modified ONs for the development of unique molecular machines and the control of various biological phenomena.

## 3. Experimental

### 3.1. General

Reagents and solvents were purchased from commercial suppliers and were used without purification unless otherwise specified. All experiments involving air and/or moisture-sensitive compounds were carried out under N_2_ or Ar atmosphere. All reactions were monitored with analytical TLC (Merck Kieselgel 60 F254). Column chromatography was carried out with a Fuji Silysia FL-100D. Physical data were measured as follows: NMR spectra were recorded on a JEOL JNM-ECS-500 spectrometer in CDCl_3_ or DMSO*-d_6_* as the solvent with tetramethylsilane as an internal standard. IR spectra were recorded on a JASCO FT/IR-4200 spectrometer. Optical rotations were recorded on a JASCO P-2200 instrument. FAB mass spectra were measured on a JEOL JMS-700 mass spectrometer.

### 3.2. Preparation of 5-(4-Phenyldiazenylphenyl)ethynyl-2'-deoxyuridine (**1**)

Under an argon atmosphere, 4-ethynylazobenzene (**3** [13], 1.06 g, 5.12 mmol), Pd(PPh_3_)_4_ (592 mg, 0.512 mmol), and CuI (113 mg,0.512 mmol) was dissolved in dry DMF (50 mL). Then, Et_3_N (3.6 mL) and 2'-deoxy-5-iodouridine (**2**, 1.81 g, 5.12 mmol) were added. The reaction mixture was stirred at 60 °C for 4 h. The resultant mixture was filtered over Celite. The filtrate was concentrated *in vacuo*. The residue was purified by silica gel column chromatography and eluted with CHCl_3_/MeOH (20:1), to give compound **1** (1.80 g, 81%) as a light-orange powder: M.p. 208–210 °C; IR (KBr): *ν* 3439 (NH, OH), 1617 (C=O), 1289 (N=N) cm^−1^; 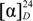
−3.7 (c 1.00, DMSO); ^1^H-NMR (500 MHz, DMSO-*d*_6_): *δ* 11.7 (1H, brs, NH), 8.47 (1H, s, H-6), 7.94–7.90 (4H , m), 7.69–7.57 (5H, m), 6.14 (1H, t, *J* = 6.5 Hz, *H-*1'), 5.27 (1H, d, *J* = 4.0 Hz, H-3'), 5.20 (1H, t, *J* = 5.0 Hz, C-H4'), 4.30–4.26 (1H, m, OH), 3.82 (1H, m, OH), 3.71–3.58 (2H, m, H-5'), 2.21–2.17 (2H, m, H_-_2'); ^13^C-NMR (125 MHz, DMSO-*d_6_*): *δ* 161.3, 151.9, 151.0, 149.4, 132.2, 131.8, 129.5, 125.4, 122.9, 122.6, 97.8, 91.5, 87.6, 85.6, 84.9, 69.8, 60.8, 40.2; FAB-LRMS *m/z* = 433 (MH^+^); FAB-HRMS calcd for C_23_H_21_N_4_O_5_ 433.1506, found 433.1524.

### 3.3. Preparation of 5'-O-(4,4'-Dimethoxytrityl)-5-(4-phenyldiazenylphenyl)ethynyl-2'-deoxyuridine (**4**)

To a solution of compound **1** (141 mg, 0.324 mmol) in dry pyridine (3 mL) was added DMTrCl (131 mg, 0.389 mmol) at room temperature, and the reaction mixture was stirred for 4 h. The reaction was quenched by the addition of MeOH with 10 min stirring. The solvent was removed *in vacuo*, and the residue was partitioned between CHCl_3_ and H_2_O. The separated organic layer was washed with H_2_O, followed by brine. The organic layer was dried (Na_2_SO_4_) and concentrated in vacuo. The residue was purified by silica gel column chromatography and eluted with CHCl_3_/MeOH (20:1 with 0.5% Et_3_N) to give Compound **4** (239 mg, 88%) as an orange foam: IR (KBr): *ν* 3437, 3410(NH, OH), 1701 (C=O), 1272 (N=N) cm^−1^; 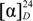 36.2 (c 1.00, CHCl_3_); ^1^H-NMR (500 MHz, CDCl_3_): *δ* 8.51 (1H, brs, NH), 8.29 (1H, s, H-6), 7.90 (2H, d, *J* = 7.5 Hz), 7.70 (2H, d, *J* = 8.5 Hz), 7.52–7.45 (5H, m), 7.37–7.28 (6H, m), 7.16 (1H, dd, *J* = 6.5 and 1.0 Hz), 7.10 (2H, d, *J* = 8.0 Hz), 6.82–6.79 (4H, m) 6.38 (1H, dd, *J* = 7.5, 6.5 Hz, H-1'), 4.60–4.59 (1H, m, H-3'), 4.14–4.13 (1H, m, H-4'), 3.70 (3H, s, OMe), 3.69 (3H, s, OMe), 3.50 (1H, dd, *J* = 8.0 and 3.0 Hz, H-5'), 3.34 (1H, dd, *J* = 8.0 and 3.0 Hz, H-5'), 2.57–2.53 (1H, m, H-2'), 2.40–2.34 (1H, m, H-2'), 2.09 (1H, brs, OH); ^13^C-NMR (125 MHz, CDCl_3_): *δ* 158.6, 152.6, 151.7, 148.8, 144.3, 135.4, 132.4, 131.3, 129.9, 129.1, 128.1, 127.9, 127.1, 125.1, 122.9, 122.5, 113.4, 100.4, 93.6, 87.2, 86.7, 85.9, 82.2, 72.4, 63.3, 55.2, 41.7; FAB-LRMS *m/z* = 757 (MNa^+^); FAB-HRMS calcd for C_44_H_38_N_4_O_7_Na 757.2633, found 757.2633.

### 3.4. Preparation of 3-O-{2-Cyanoethyl(diisopropylamino)phosphino}-5'-O-(4,4'-Dimethoxytrityl)-5-(4-phenyldiazenylphenyl)ethynyl-2'-deoxyuridine (**5**)

To a solution of compound **4** (188 mg, 0.26 mmol) in dry MeCN (5 mL) was added *N, N*-diisopropylamine (0.13 mL,0.76 mmol) and 2-cyanoethyl-*N, N'*-diisopropylchlorophosphoramidite (0.09 mL, 0.40mmol) at room temperature, and the reaction mixture was stirred for 1.5 h. The resultant mixture was partitioned between AcOEt and H_2_O. The separated organic layer was washed with saturated aqueous NaHCO_3_, followed by brine. The organic layer was dried (Na_2_SO_4_) and concentrated in vacuo. The residue was purified by silica gel column chromatography and eluted with CHCl_3_/MeOH (20:1 with 0.5% Et_3_N), to give a 17:3 diastereomeric mixture of **5** (324 mg, 82%) as an orange foam: IR (KBr): *ν* 3610 (NH), 1699 (C=O), 1272 (N=N) cm^−1^; 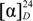 32.5 (c 1.00, CHCl_3_); ^1^H-NMR (500 MHz, CDCl_3_): *δ* 9.08 (1H, brs, NH), 8.35 (0.85H, s, H-6), 8.30 (0.15H, s, H-6), 7.89 (2H, d,*J* = 7.5 Hz), 7.67 (2H, d, *J* = 8.5 Hz), 7.55–7.04 (14H, m), , 6.67–6.75 (4H, m), , 6.35 (1H, dd, *J* = 7.5, 6.0 Hz, H-1'), 4.68–4.61 (1H, m, H-3'), 4.26 (1H, m, H-4'), 3.70 (3H, s, OMe), 3.69 (3H, s, OMe), 3.67–3.53 (5H, m, CH_2_CH_2_CN, H-5'), 3.31 (1H, dd, *J* = 8.5, 2.5 Hz, H-5'), 2.65–2.56 (1H, m, H-2'), 2.47–2.36 (3H, m, H-2', ((CH_3_)_2_C*H*)_2_N), 1.18 (12H, d, *J* = 6.5 Hz, ((C*H*_3_)_2_CH)_2_N); ^13^C-NMR (125 MHz, CDCl_3_): *δ* 161.2, 158.5(9), 158.5(6), 152.6, 151.5, 149.1, 144.35, 142.5, 135.4, 132.3, 132.0, 131.1, 130.0 (d, *J* (C, P) = 6.0 Hz), 129.1, 128.7, 128.0, 127.9 ,127.0, 125.1, 122.8, 122.4, 120.5, 117.3, 113.3, 100.3, 93.4, 86.3 (d, *J* (C, P) = 3.5 Hz), 85.9, 82.4, 77.3, 77.0, 76.8, 73.4, 73.2, 63.0, 58.2, 58.1, 55.1, 43.2 (d, *J* (C, P) = 13.0 Hz), 40.8 (d, *J* (C, P) = 5.0 Hz), 25.6, 24.5(9), 24.5(3), 24.4(8), 20.2 (d, *J* (C, P ) = 7.0 Hz); ^31^P-NMR (200 MHz, CDCl_3_): *δ* 149.09, 148.66; FAB-LRMS *m/z* = 957 (MNa^+^); FAB-HRMS calcd for C_53_H_5__5_N_6_O_8_PNa 957.3711, found 957.3711.

### 3.5. Synthesis of **dU^Az^**-Modified Oligodeoxynucleotides

Solid-phase oligonucleotide synthesis was performed on an nS-8 Oligonucleotides Synthesizer (GeneDesign, Inc., Osaka, Japan) using commercially available reagents and phosphoramidites with 5-(bis-3, 5-trifluoromethylphenyl)-1*H*-tetrazole (0.25 M concentration in acetonitrile) as the activator. **dU^Az^** phosphoramidite was chemically synthesized as described above. All of the reagents were assembled, and the oligonucleotides were synthesized according to the standard synthesis cycle (trityl on mode). Cleavage from the solid support and deprotection were accomplished with concentrated ammonium hydroxide solution at 55 °C for 12 h. The crude oligonucleotides were purified with Sep-Pak Plus C18 cartridges (Waters) followed by RP-HPLC on a XBridge^TM^ OST C18 Column, 2.5 μm, 10 × 50 mm (Waters) using MeCN in 0.1 M triethylammonium acetate buffer (pH 7.0). The purified oligonucleotides were quantified by UV absorbance at 260 nm and confirmed by MALDI-TOF mass spectrometry (Table 4).

**Table 4:** Yields and MALDI-TOF MS data of **dU^Az^**-modified oligonucleotide.

Oligodeoxynucleotide		Yield	MALDI-TOF MS	
			Calcd. [M-H]^−^	found [M-H]^−^
5'-d(GCGTT**U^Az^**TTTGCT)-3'	**7**	29%	3822.6	3822.4

### 3.6. UV Melting Experiments

Melting temperatures (*T*_m_) were determined by measuring the change in absorbance at 260 nm as a function of temperature using a Shimadzu UV-Vis Spectrophotometer UV-1650PC equipped with a *T*_m_ analysis accessory TMSPC-8. Equimolecular amounts of the target DNA/RNA and oligonucleotides were dissolved in 10 mM sodium phosphate buffer (pH 7.0) containing 100 mM NaCl to give a final strand concentration of 4.0 µM. The melting samples were denatured at 100 °C and annealed slowly to room temperature. Absorbance was recorded in the forward and reverse directions at temperatures of 5 to 90 °C at a rate of 0.5 °C/min.

### 3.7. Photoisomerization of **dU^Az^**

The *trans-*to-*cis* isomerization was performed with a UV-LED lamp (ZUV-C30H; OMRON) and a ZUV-L10H lens unit (760 mW/cm^2^). The *cis*-to-*trans* isomerization was performed with a Xenon lamp (MAX-303; Asahi Spectra Co., Ltd., Tokyo, Japan) and XHQA420 optical filter. Absorbance spectra of *trans-cis* ON **7** were measured by a Shimadzu UV-Vis Spectrophotometer UV-1650PC. Conditions: ON **7** (4.0 µM), NaCl (100mM) in sodium phosphate buffer (10 mM, pH 7.0).

## 4. Conclusions

We have synthesized a new photoisomeric nucleoside, C5-azobenzene-modified 2'-deoxyuridine **dU^Az^** using Sonogashira-type cross-coupling as a key step. **dU^Az^** showed very rapid reversible *cis-trans* photoisomerization with monochromic light at the appropriate wavelength in oligodeoxynucleotide. **dU^Az^**-modified oligodeoxynucleotide showed an interesting duplex-forming property, namely, the *T*_m_ values of both the **dU^Az^**-modified ON/DNA and **dU^Az^**-modified ON/RNA were higher for the cis-form than for the *trans*-form, unlike conventional azobenzene-modified ONs. Additionally, it was revealed that installation of **dU^Az^** into oligodeoxynucleotide had little influence on the mismatch recognition ability.

## Supplementary Materials

Supplementary materials can be accessed at: http://www.mdpi.com/1420-3049/19/4/5109/s1.
