# Supplementary material for: C5-Azobenzene-substituted 2'-Deoxyuridine-containing Oligodeoxynucleotides for Photo-Switching Hybridization
Source: Molecules. 2014 Apr 22;19(4):5109–18. doi: 10.3390/molecules19045109 (PMC6271114; doi:10.3390/molecules19045109)

Supplementary Information

1. ^{1}H, ^{13}C and ^{31}P Spectra of New Compounds

Figure S1. ^{1}H-NMR spectrum of compound **1**.

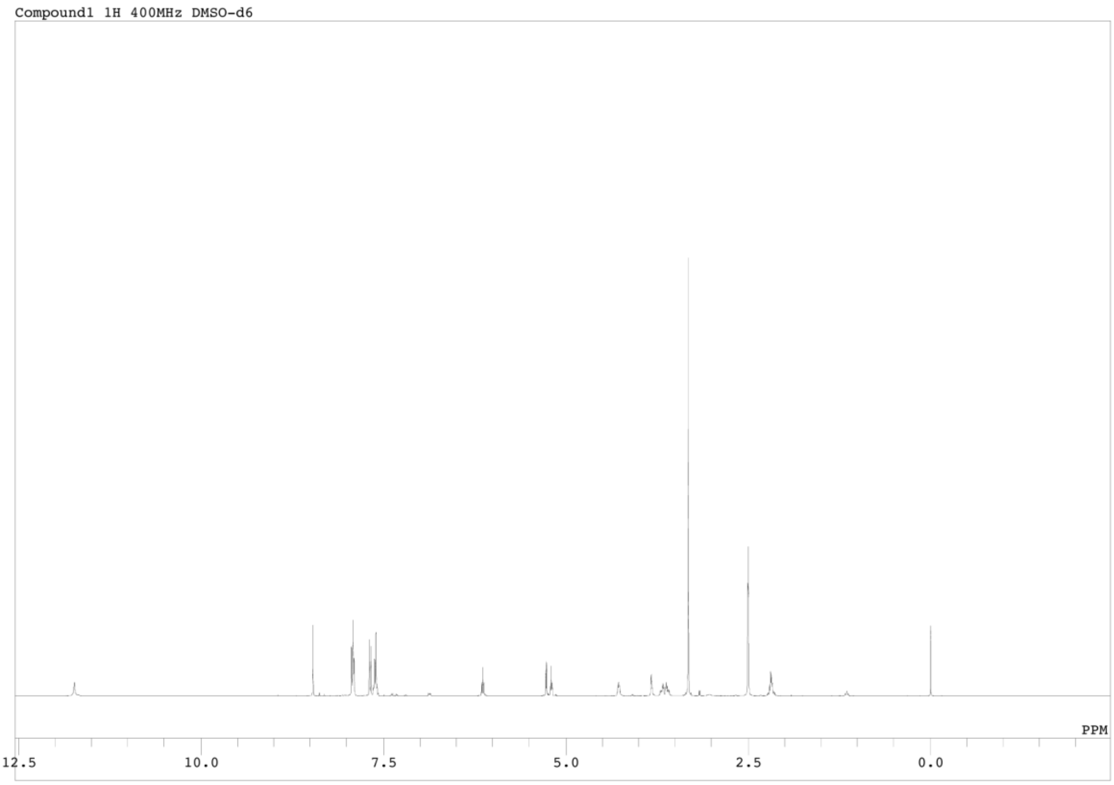

Figure S2. ^{13}C-NMR spectrum of compound **1**.

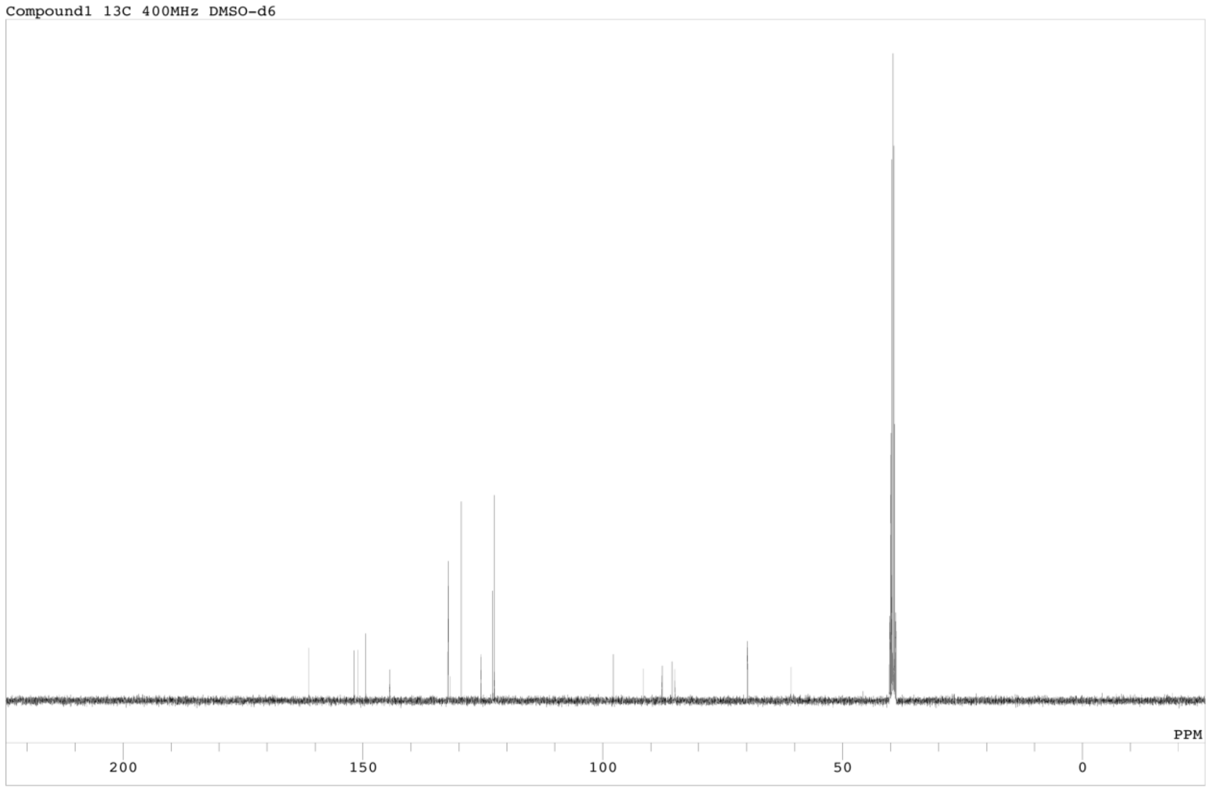

Figure S3. ^{1}H-NMR spectrum of compound **4**.

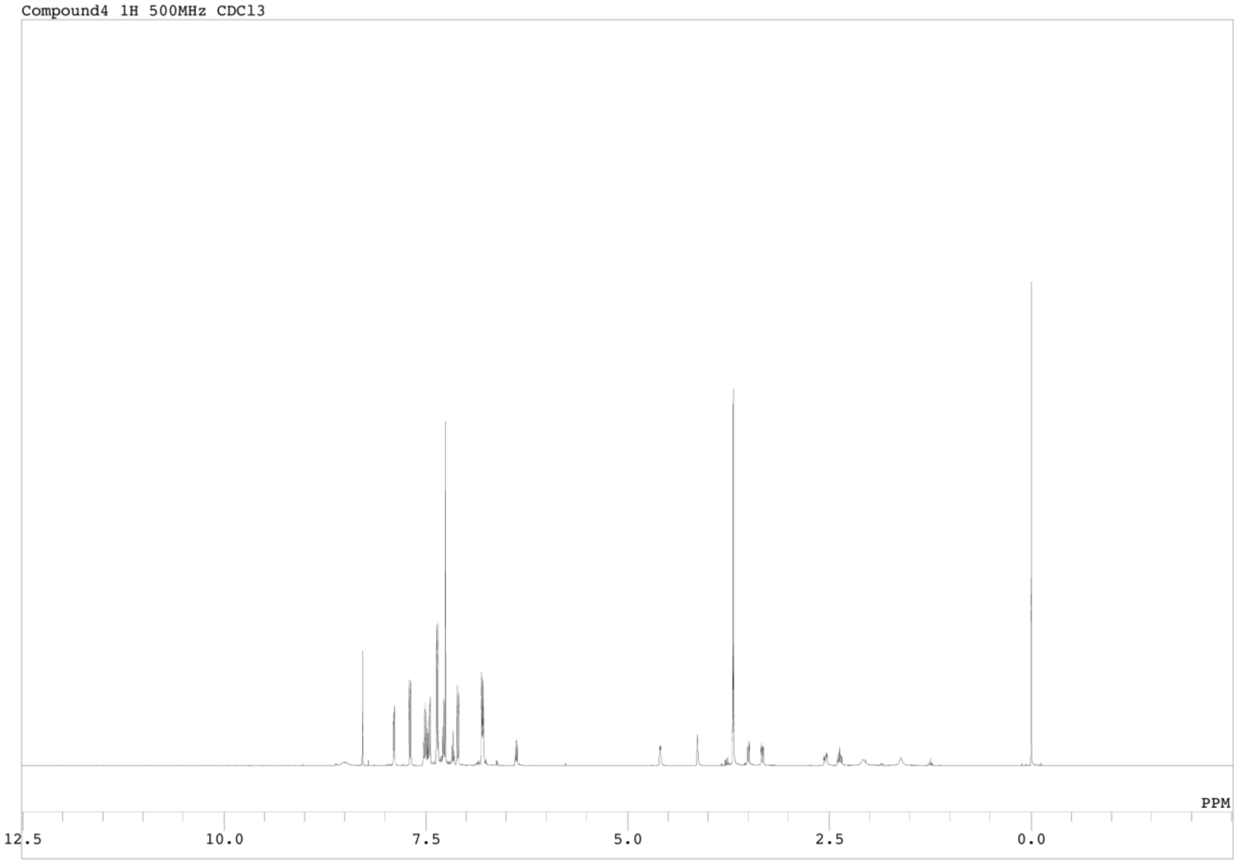

Figure S4. ^{13}C-NMR spectrum of compound **4**.

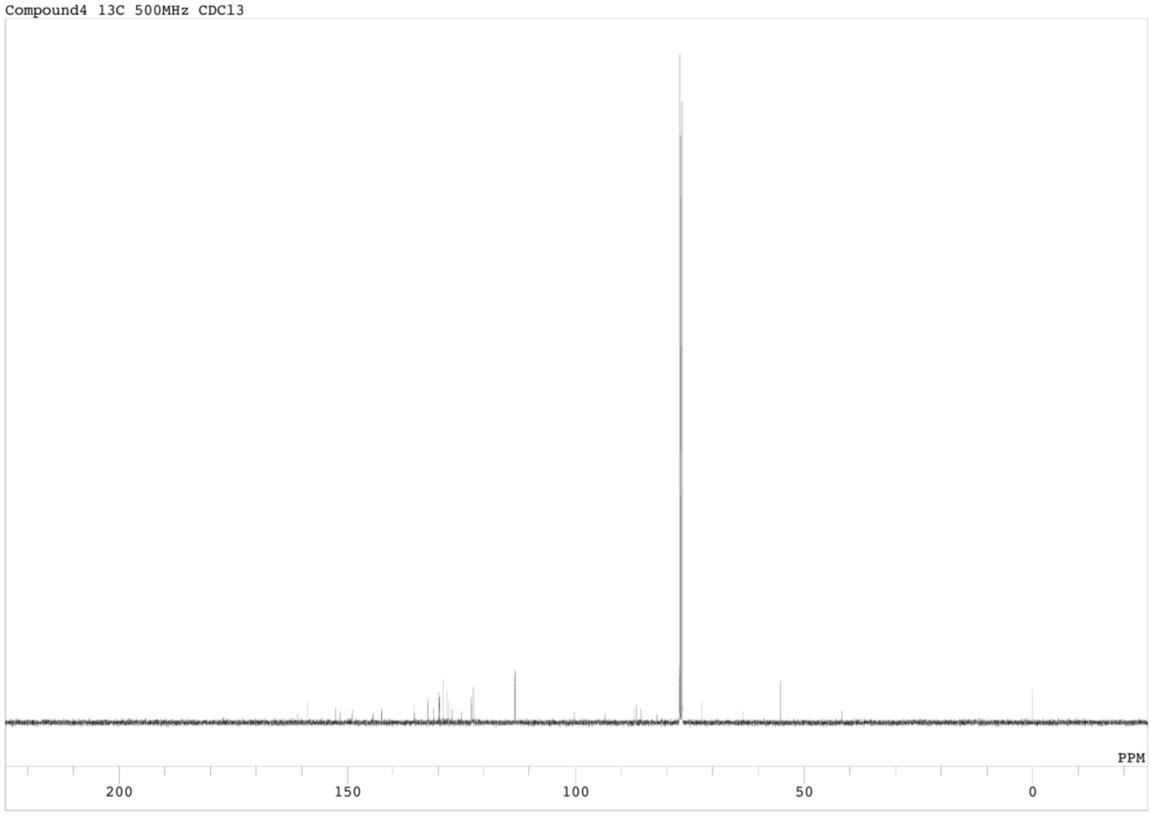

Figure S5. ^{1}H-NMR spectrum of compound **5**.

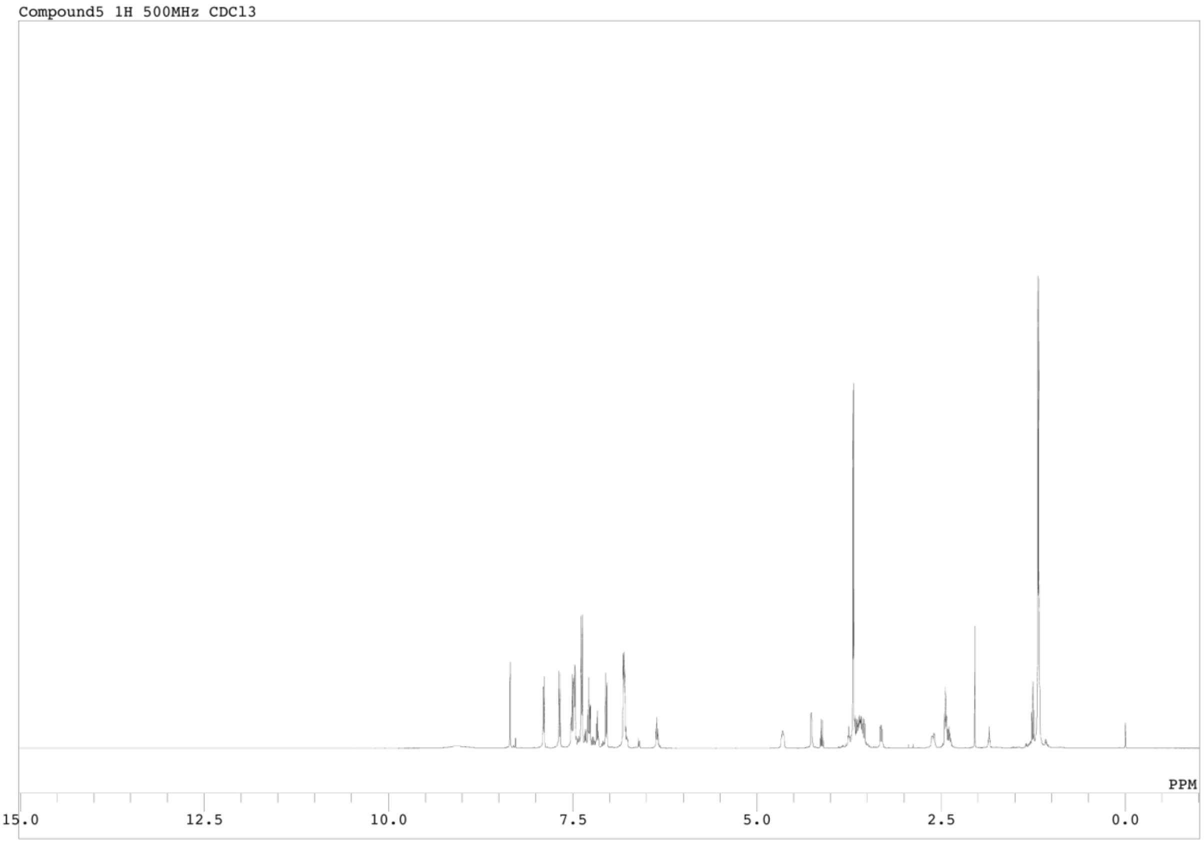

Figure S6. ^{13}C-NMR spectrum of compound **5**.

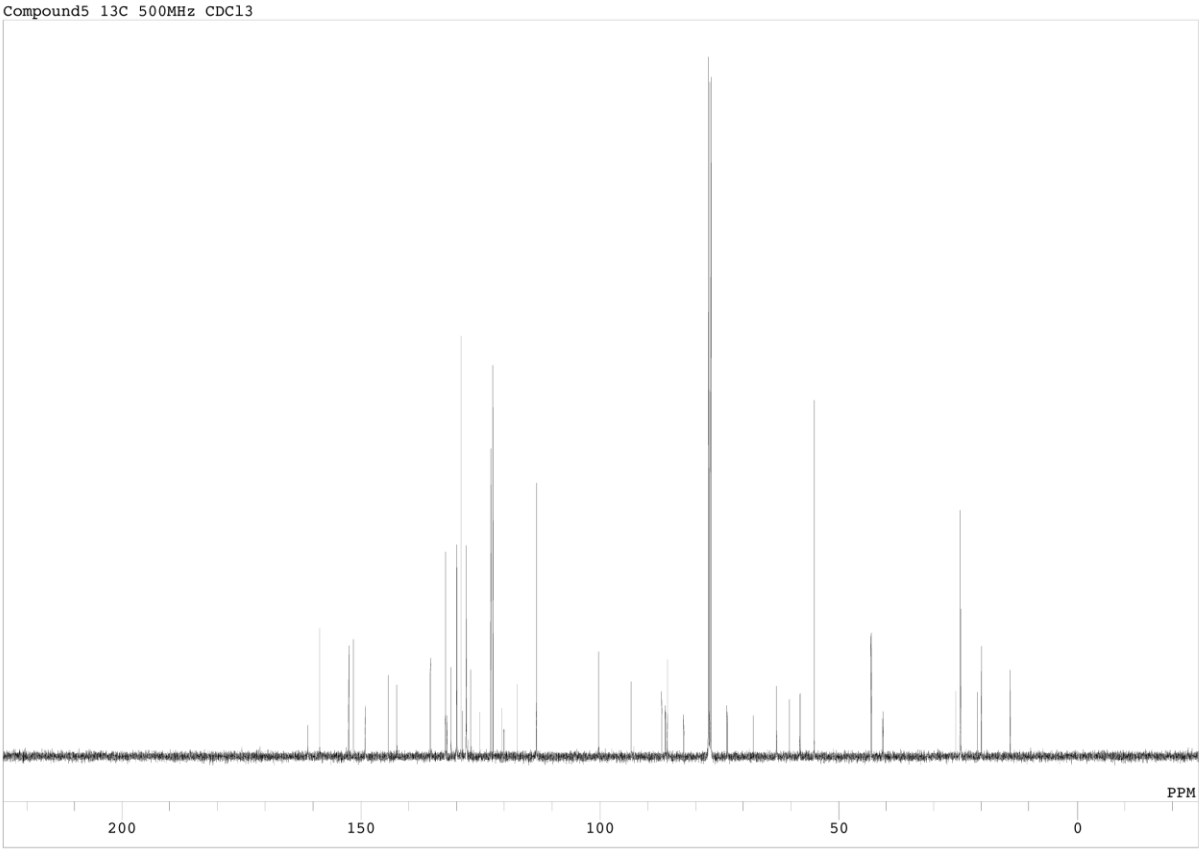

Figure S7. ^{31}P-NMR spectrum of compound **5**.

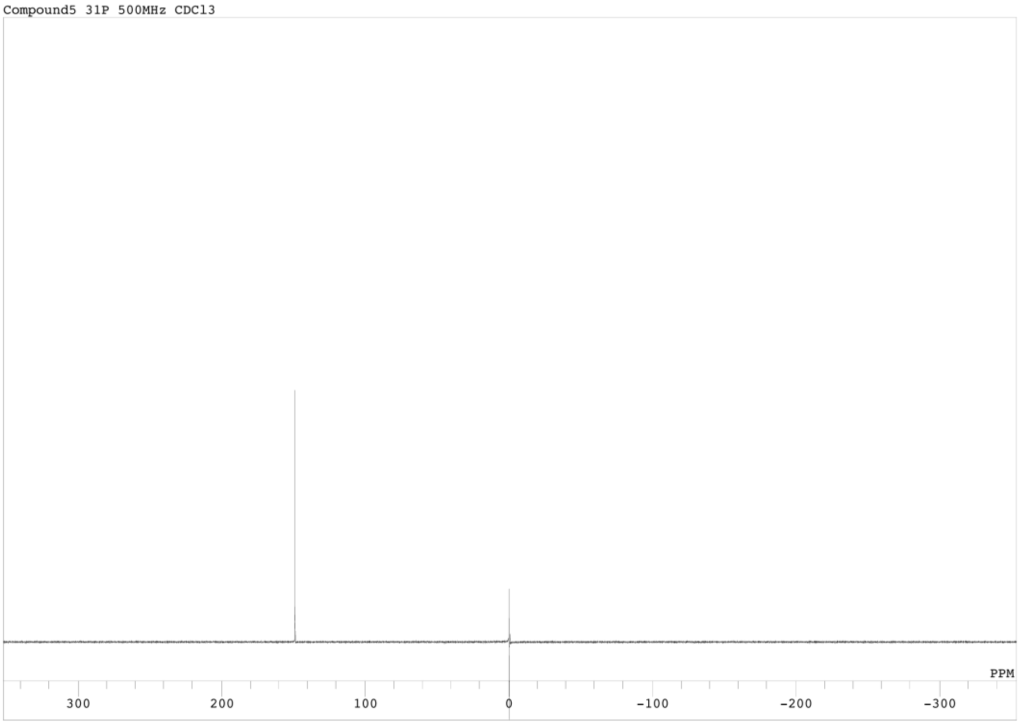

2. HPLC and MALDI-TOF MS Analysis of dU^{Az}-Modified Oligodeoxynucleotide

ON **7**

HPLC

Column: Waters XBridge™ OST C18 2.5 μm, 4.6 × 50 mm

Gradient: 10%–20% MeCN (over 30 min) in triethylammonium acetate buffer (pH 7.0, 0.1 M)

Flow rate: 1.0 mL/min

Column temperature: 50 ℃

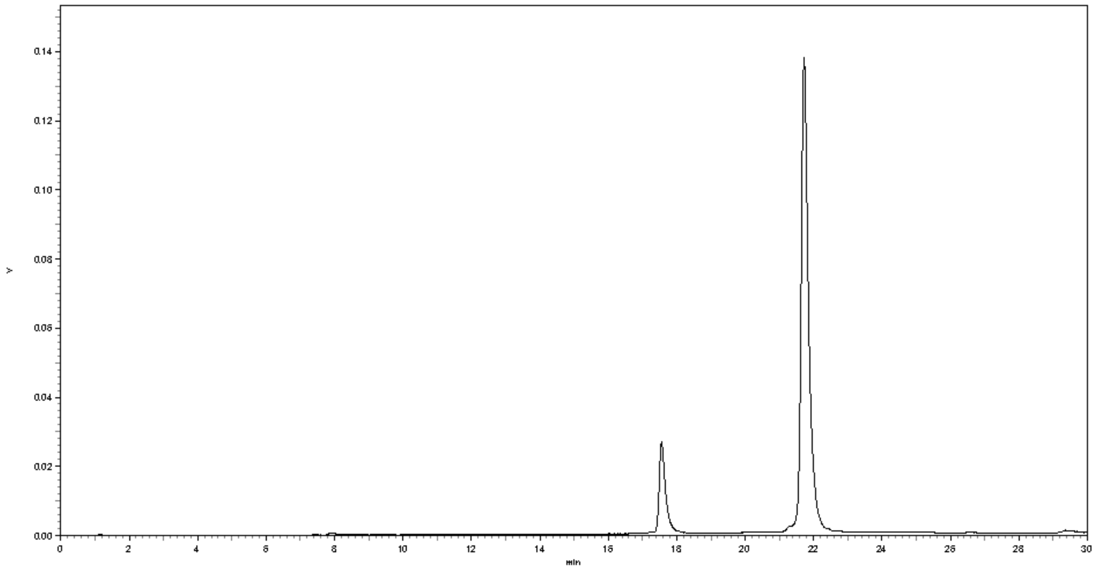

MALDI-TOF MS

Calcd. 3822.6 [M−H]$^-$

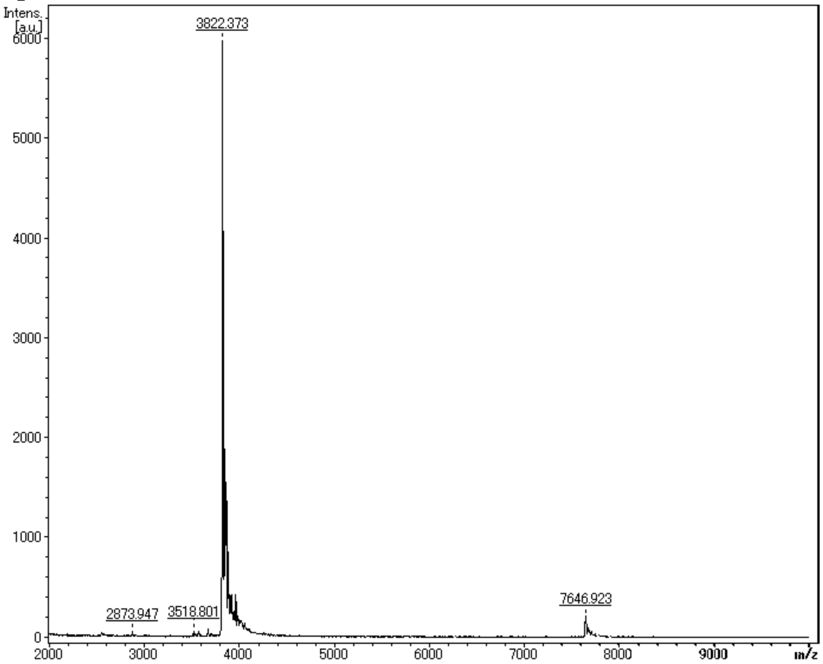

3. UV-Melting Points of DNA/RNA Duplexes with a Mismatched Base Pair

Table S1. UV-melting points [℃] of DNA/RNA duplexes. [a]

Duplex	Bases	T_m [℃]		ΔT_m [℃] [b]	
		Trans [c]	***Cis*** [d]	***Trans*** [c]	***Cis*** [d]
**6/13**	T:U	30		−17	
**6/14**	T:C	29		−18	
**6/15**	T:G	37		−10	
**7/13**	**U^{Az}**:U	29	30	−13	−17
**7/14**	**U^{Az}**:C	26	29	−11	−18
**7/15**	**U^{Az}**:G	34	37	−8	−10

[a] All T_m values for the duplexex (4.0 μM) were determined in 10 mM sodium phosphate buffer (pH 7.0) containing 100 mM NaCl. The T_m values given are the average of at least three data points; [b] ΔT_m values are calculated relative to the T_m values of matched DNA **6**/RNA **12** (47 ℃) or ON **7**/RNA **12** (42 ℃ for *trans* and 47 ℃ for *cis*) duplexes; [c] The percentage of *trans* isomer was *ca.* 80%; [d] The percentage of *cis* isomer was *ca.* 60%.

4. UV Melting Curves of dU^{Az}-Modified Duplexes

Figure S8. UV melting curves for the duplexes formed between *cis*- (red line), *trans*- (black line) ON **7** and ON **8–15**.

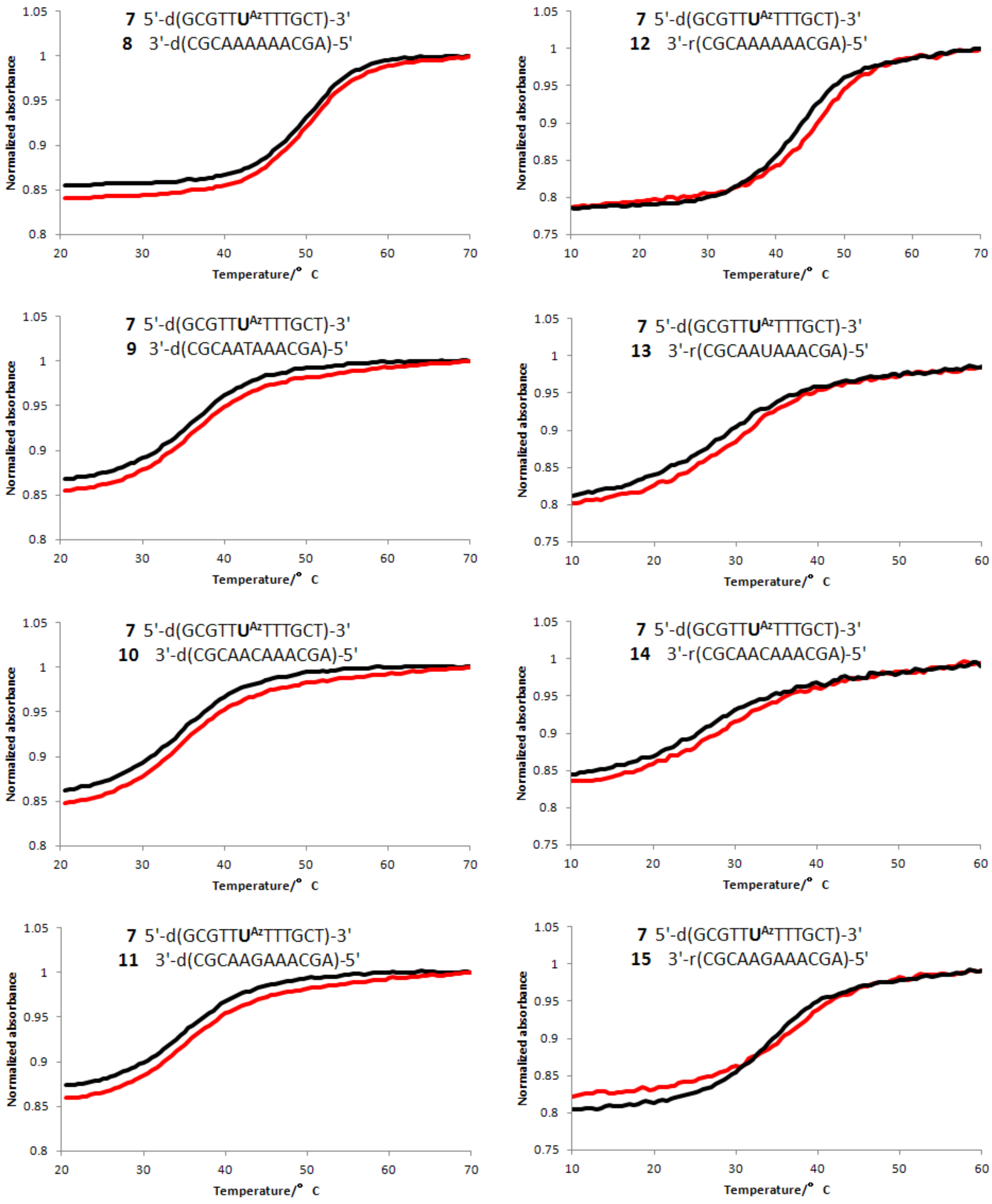

Supplement: Supplementary file 1 [file molecules-19-05109-s001.pdf]
